# Upper Crossed Syndrome and Scapulae Upper-Trapping: A Mesotherapy Protocol in Cervicoscapulobrachial Pain—The 8:1 Block

**DOI:** 10.3390/bioengineering11111142

**Published:** 2024-11-13

**Authors:** Luyddy Pires, Napoliane Santos, João Vitor Lana, Alex Pontes de Macedo, Fábio Ramos Costa, Gabriel Ohana Marques Azzini, Tomas Mosaner, Daniel de Moraes Ferreira Jorge, Gabriel Silva Santos, Arthur Medeiros, José Alexandre Reale Pereira, José Fábio Lana

**Affiliations:** 1 Orthopedics, Brazilian Institute of Regenerative Medicine (BIRM), Indaiatuba 13334-170, SP, Brazil; luyddypires@gmail.com (L.P.); dranapolianesantos@gmail.com (N.S.); alex_macedo@icloud.com (A.P.d.M.); drgabriel.azzini@gmail.com (G.O.M.A.); tmosaner@uol.com.br (T.M.); danfjorge@gmail.com (D.d.M.F.J.); josefabiolana@gmail.com (J.F.L.); 2 Regenerative Medicine, Orthoregen International Course, Indaiatuba 13334-170, SP, Brazil; 3 Medical School, Max Planck University Center (UniMAX), Indaiatuba 13343-060, SP, Brazil; jvblana@gmail.com; 4 Orthopedics, FC Sports Traumatology, Salvador 40296-210, BA, Brazil; fabiocosta123@uol.com.br; 5 Orthopedics, Ortocentro, Recife 51110-160, PE, Brazil; arthurmedeiros@me.com; 6 Orthopedics, Madre Teresa Hospital, Belo Horizonte 30441-070, MG, Brazil; sou@hospitalmadreteresa.org; 7 Clinical Research, Anna Vitória Lana Institute (IAVL), Indaiatuba 13334-170, SP, Brazil; 8 Medical School, Jaguariúna University Center (UniFAJ), Jaguariúna 13911-094, SP, Brazil

**Keywords:** upper crossed syndrome, scapular dyskinesis, cervicoscapulobrachialgia, mesotherapy protocol, scapular upper trapping

## Abstract

Upper Crossed Syndrome (UCS), described by Vladimir Janda, is characterized by postural changes involving the cervical spine and trunk, leading to biomechanical limitations and cervicoscapulobrachial pain. This study proposes a mesotherapy protocol, termed the 8:1 block, to address cervicoscapulobrachialgia by targeting the scapulae and associated musculature. The scapula, central to shoulder girdle kinematics, often exhibits dyskinesis and muscular imbalances, notably the pattern referred to as scapular upper trapping (SUT). SUT involves scapular elevation, medial rotation, and shoulder protraction, contributing to cervicobrachial pain. The protocol includes a comprehensive assessment of muscle tone changes and biomechanical considerations, highlighting the importance of the scapula in upper limb movement and posture. Key anatomical changes involve tightened upper trapezius, levator scapulae, and pectoralis minor muscles, with weakened middle trapezius and serratus anterior. The mesotherapy approach targets these imbalances through specific injection points to alleviate muscle tension and correct postural deviations. Case studies from our clinic demonstrate the protocol’s effectiveness in reducing pain and restoring scapular biomechanics. Patients reported significant improvements in pain relief and functional outcomes, underscoring the clinical utility of the 8:1 block in treating cervicoscapulobrachialgia. This protocol offers a feasible, cost-effective intervention that enhances the efficacy of traditional therapeutic exercises by addressing underlying muscular and biomechanical dysfunctions. In conclusion, the 8:1 block mesotherapy protocol provides a novel approach to managing cervicoscapulobrachial pain by focusing on scapular biomechanics and muscle tension. Further studies are needed to validate these findings and refine the protocol for broader clinical application.

## 1. Introduction

**Figure 1:**
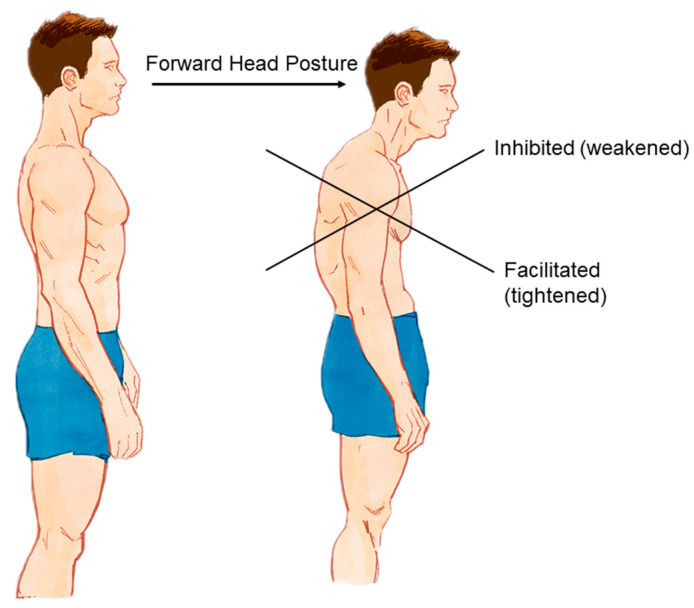
Postural change of the UCS leading to FHP. A normal posture (**left**) compared with the altered posture associated with UCS (**right**), characterized by FHP and a cross pattern of muscle imbalances. The inhibited (weakened) muscles are indicated by the upper arrows, and the facilitated (tightened) muscles are shown by the lower arrows. These postural changes contribute significantly to cervicoscapulobrachial pain and dysfunction.

Described by Vladimir Janda, the Upper Crossed Syndrome (UCS) is characterized by a syndromic postural change that involves the cervical spine and trunk [1] (Figure 1). Being multifactorial, this alteration culminates in a cycle of variations in muscle tone, which lead to a postural pattern called forward head posture (FHP), already studied in detail since the beginning of the 20th century [1,2,3]. Widely noticed in patients with painful syndromes in the proximal region of the body in the transverse plane, this pattern imposes biomechanical and kinesiological limitations that require therapeutic intervention [4].

Upper Crossed Syndrome (UCS) is prevalent in individuals of varying ages, but it is often more pronounced in older adults due to age-related degeneration of muscles, posture, and joint flexibility. Younger individuals may experience UCS as a result of prolonged poor posture (e.g., from desk work or mobile device use), while in older adults, the severity may be compounded by muscular atrophy, reduced mobility, and degenerative changes in the spine [4].

Although several conventional treatments for Upper Crossed Syndrome (UCS) exist—such as physical therapy, manual therapy, and pharmacological interventions—this technical note focuses on introducing the 8:1 block mesotherapy protocol as a novel treatment option. Traditional approaches tend to manage symptoms, whereas our protocol directly targets muscle imbalances and postural deviations.

The 8:1 block mesotherapy protocol is a new treatment approach specifically targeting muscular imbalances associated with Upper Crossed Syndrome. While mesotherapy as a technique has been used in other contexts, its application to UCS and the specific injection points described in this protocol are novel. Future clinical trials are needed to validate this approach for broader use.

**Figure 2:**
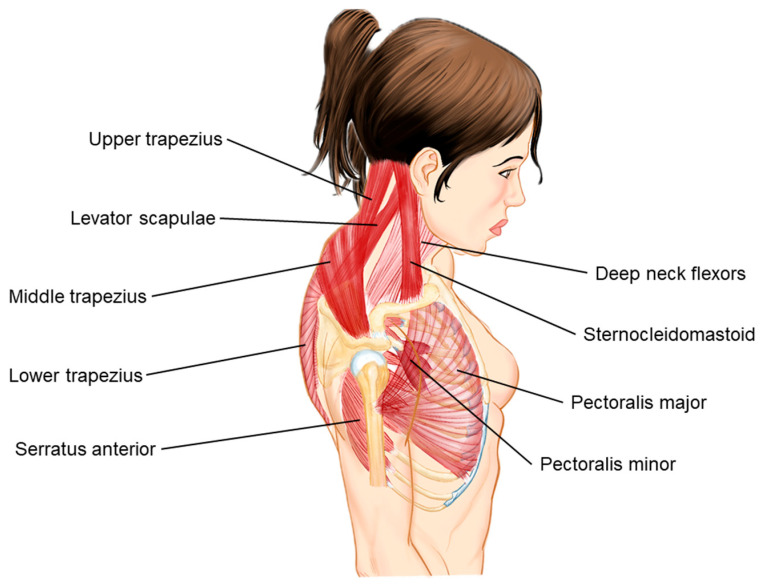
Muscle imbalances in UCS. The labeled muscles, related to the UCS, include the upper trapezius, levator scapulae, middle trapezius, lower trapezius, serratus anterior, deep neck flexors, sternocleidomastoid, pectoralis major, and pectoralis minor. The image highlights the typical postural distortion seen in UCS, where the upper trapezius and levator scapulae (dark red) are often overactive and tight, while the deep neck flexors and lower trapezius are typically weak (light red). These imbalances build the dysfunction and pain in the cervicoscapulobrachial region.

The changes in muscle tone as mentioned above and the muscular actions are explained in Table 1 and Figure 2. Understanding this postural pattern is important to evaluate the possible pathological causes and the targets of therapeutic intervention. Similarly, the inspectional assessment of the effectiveness of the treatment consists of the immediate correction of this postural pattern.

## 2. Biomechanical and Kinesiological Considerations

In the biomechanical core of this intersection, the scapula is a flat bone that plays a central role in the kinematics of the shoulder girdle, an anatomical conglomerate that encompasses it together with the clavicle and sternal manubrium [5]. Forming three distinct joints with different functions, which are the sternoclavicular, acromioclavicular and scapulothoracic joints, the shoulder girdle works with peculiar biomechanics regarding the movement of the upper limbs. About the scapula itself, the usual movements are elevation and depression in the coronal plane; protraction and retraction in the sagittal plane; and medial and lateral rotations in the transverse plane [6,7]. Regarding its relationship with the humerus and upper limb, there is what is called scapulohumeral rhythm. For every two degrees of range of motion of the glenohumeral (GU) joint, the scapulothoracic joint responds with one degree of movement, bringing a ratio of 2:1. This association seeks to maintain the length–tension relationship of the shoulder muscles for greater efficiency and to protect the GU joint from impacts against anatomical structures [6,7].

When there is a break in this movement pattern, due to loss of arthrokinematic control, what is called scapular dyskinesis happens. If not corrected, this altered pattern can result in changes in the tone of the shoulder girdle muscles and lead to very symptomatic musculoskeletal pathologies [7].

**Figure 3:**
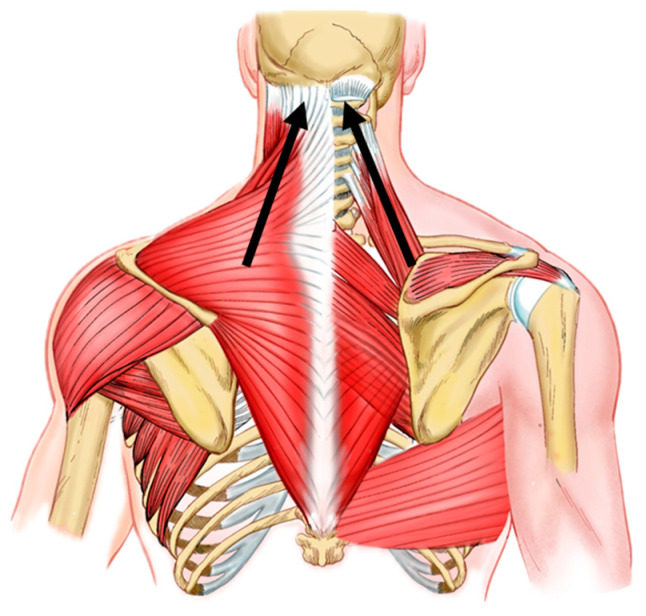
Tension lines in UCS. The usual vectors of tension in UCS are indicated by black arrows. Upper trapezius and levator scapulae muscles are often tightened and overactive in UCS. These tension lines represent the typical patterns of muscle imbalance and postural distortion seen in UCS, contributing to cervicoscapulobrachial pain and leading to the SUT definition. Understanding these tension lines is crucial for effectively targeting therapeutic interventions.

This anomalous pattern of scapular myotendinous structures leads to a pattern that we will henceforth call scapular upper trapping (SUT) (Figure 3). This is characterized, in a homologous way to UCS, by the elevation and medial rotation of the scapula with shoulder protraction.

## 3. Cervicoscapulobrachialgia: A Proposition

Therefore, since the scapula is the link that unites the trunk to the upper limb, we hypothesized that it could be part of the differential diagnosis of cervicobrachial syndromes. We thus propose the term cervicoscapulobrachialgia to designate painful conditions that have the scapula as a fundamental part of their genesis. Considering that, mainly, the muscle groups that participate in the elevation of the scapula have their origin in the base of the skull and cervical spine [5], tonic and biomechanical changes in this functional core may be related to the classic symptoms of cervicobrachialgia, in addition to tension headaches (Figure 3).

## 4. Differential Diagnoses in Cervicoscapulobrachialgia

Thus, it is important that the most common differential diagnoses are investigated during patient assessment. The physical examination should cover everything from the base of the skull to the muscles involved in flexion–extension of the elbow. Objectively, we suggest standardizing the physical examination into three stages: specific and targeted postural assessment, myofascial palpation of anatomical landmarks and special musculoskeletal tests. This assessment must be multiparametric, making use of auxiliary diagnostic instruments, such as simetograph, goniometer, ultrasound, and thermography. It is important to remember that defining the target for intervention is the most important point in medical and multidisciplinary workup, as the treatment will never achieve the ideal result if the approach is inefficient.

In a craniocaudal assessment, the most cranial condition that involves the myofascial system in this region studied is tension headache. This is considered a primary headache that has a neurobiological basis, triggered by central factors, but with reflexes in the entheses of the skull base muscles [8]. Going down to the cervical region, neck pain is irregularly studied, without a satisfactory diagnostic target, although it is quite prevalent throughout the world population [9]. Possible diagnoses for this syndromic presentation are emotional and/or psychological factors, neuromuscular disorders, degenerative diseases (autoimmune, genetic) and postural changes [10]. Presentations with cervicobrachial irradiation are not so clear in diagnosis, being of neurogenic origin in only around 20% of cases [11], with diagnostic possibilities being degenerative diseases of the intervertebral disc, facet degeneration, spondylolisthesis, and changes in muscular balance [11,12]. These changes in muscular balance can also lead to ligament overload of stabilizing structures.

Assessing the shoulder girdle, we propose that the scapula should be examined more carefully. Being the origin and insertion of important muscles for the movement of the upper limbs, diagnoses that should be investigated are muscle tendinopathies with entheses in the scapula and joint degeneration of the three joints that directly involve it.

**Figure 4:**
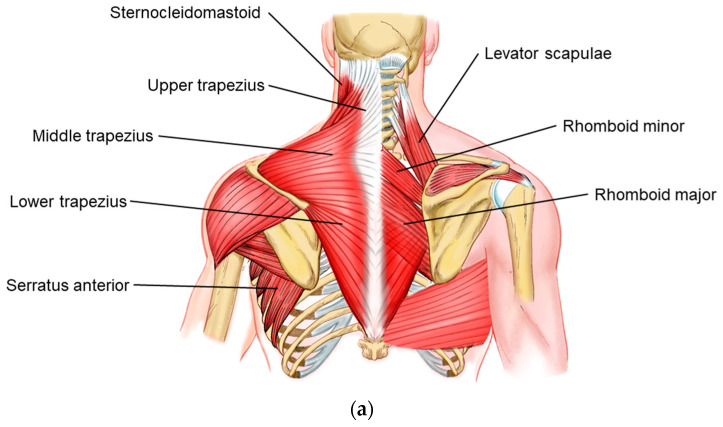

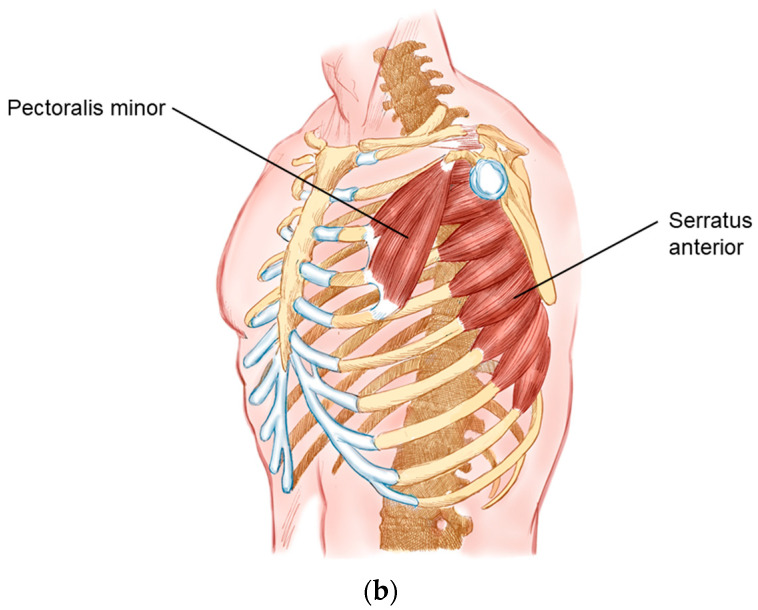
(**a**) Muscles involved in UCS and SUT. This illustration depicts the key muscles involved in the pathology of UCS and scapulae upper-trapping. The labeled muscles include the sternocleidomastoid, levator scapulae, upper trapezius, middle trapezius, lower trapezius, serratus anterior, supraspinatus, rhomboid minor, rhomboid major, and latissimus dorsi. The image highlights both the superficial and deep muscles contributing to the characteristic muscle imbalances and postural distortions seen in UCS, which are often associated with cervicoscapulobrachial pain. This context is crucial for understanding the mesotherapy protocol proposed in the 8:1 block treatment approach. (**b**) Muscles Involved in Scapular Movement and Stabilization. This illustration highlights the pectoralis minor and serratus anterior muscles, which play crucial roles in scapular movement and stabilization. The tightening of the pectoralis minor is often implicated in the protraction and internal rotation of the scapula, contributing to the muscle imbalances seen in UCS. The serratus anterior is essential for proper scapular motion and stability, and its weakness is commonly observed in UCS.

Tendinopathies of the scapular border can involve the muscles that originate from the scapula (deltoid, supraspinatus, infraspinatus, triceps, teres major and minor, coracobrachialis, biceps brachii, subscapularis and homohyoid) and those that attach to the scapula (trapezius, elevator rhomboid scapula minor and major, serratus anterior and pectoralis minor) [5]. It is worth mentioning that the pectoralis minor, originating from the coracoid process of the scapula [5], plays a relevant role in stabilizing the scapula and its mobility, and can bring with it enthesopathies and nerve compressions [13,14]. In general, it is prudent that, at least in the palpatory phase of the physical examination, these muscles are evaluated, focusing on painful points that may indicate physiological or mechanical changes. The anatomic correlation can be evaluated in Figure 4. Methodologically, the literature does not seem to concisely describe upper limb tendinopathies other than rotator cuff and elbow tendinopathies [15,16,17,18,19,20,21,22].

Like tendons, the scapular joints must undergo clinical evaluation. Of the three joints that surround the scapula, the scapulothoracic joint is the most neglected. Studied, generally by physiotherapists, for many decades [23], this joint allows variation in the shoulder’s range of motion, with the so-called scapulohumeral rhythm [6,7]. Changes in this movement pattern need to be evaluated in the patient to define the therapeutic target. The muscles involved in this process are illustrated in Figure 4a,b.

However, this joint does not respond individually to kinetic stimuli, with the sternoclavicular and acromioclavicular joint having important functions in scapular mobility, accounting for a substantial fraction of scapulocostal mobility, especially in posterior rotation actions [24].

## 5. Diagnostic Reasoning

Considering all the possibilities discussed above, there are some very important tools that help in the accurate diagnosis of the syndromic core of cervicoscapulobrachialgia. Among them, those used routinely by the authors are thermography, ultrasound, and, in selected cases, nuclear magnetic resonance.

Currently, there is a lack of evaluation of imaging patterns of the scapula, probably due to a lack of attention to the aforementioned tendinopathies and attention focused on the more common pathologies of the shoulder girdle. Therefore, bringing into discussion these possible neglected pathologies can increase the efficiency of clinical and interventional treatments involving the upper limb.

**Figure 5:**
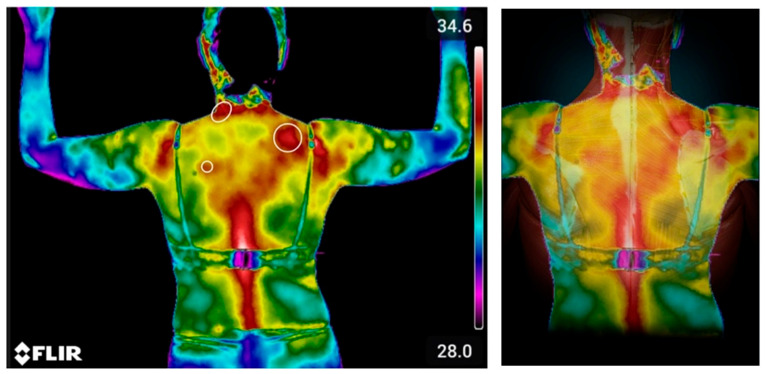
Thermography applied to UCS diagnosis. Thermography shows hyper or hypo radiation spots. Inflammatory spots are often hypervascularized and the increased blood flow leads to hypercaptation in the images. These images correlate the hypercaptation spots on the scapula levator insertion, medium trapezius and rhomboid minor.

Thermography applied to these processes under discussion is quite effective (Figure 5). It has relevant accuracy for the diagnostic investigation of tendinopathies, whose changes are noted, in these cases, by hyper-radiation in a very efficient way [25,26]. By making a clinical-imaging correlation, this is a cheap tool that adds to clinical reasoning.

**Figure 6:**
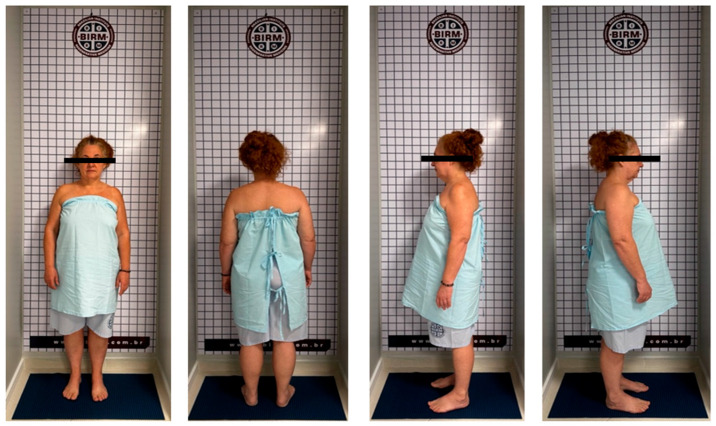
Simetograph and its application to UCS diagnosis. The simetograph is a simple device, a squared background that facilitates the visual identification of postural asymmetries. The images show a typical FHP and shoulder asymmetry (right shoulder higher).

In addition to clinical postural assessment, the simetograph helps in considering profiles of asymmetries and postural syndromes in order to quantify abnormalities (Figure 6) [6,27]. Its routine use is simple in clinical practice and can be complemented by electronic software with image capture using a simple camera [28,29].

Still, in routine clinical evaluation, ultrasound is a practical, radiation-free, and dynamic tool. Widely used to evaluate muscles, tendons and fascia, there are still no efficient protocols for standardizing musculotendinous evaluation as discussed in this article. When it comes to the shoulder girdle, there are studies that standardize the assessment of the most common pathologies [30,31], therefore not being comprehensive in musculoskeletal diagnostic investigation. It is also worth mentioning that the use of ultrasound in the office allows guided deep neuromyofascial blocks to be performed. These increase the clinical efficiency of diagnostic elucidation.

Nuclear magnetic resonance has its application in investigations by dubious clinics and as a gold standard in the evaluation of muscular constitution [32]. It then ends up being excessively expensive, encouraging clinical evaluation using other methods.

## 6. A New Protocol: The 8:1 Block

Therefore, based on the assumption that cervicoscapulobrachial pain syndrome (CSBPS) may have the scapula at its core, we propose a targeted mesotherapy technique. The therapy involves eight anesthetic blocking points in the form of mesotherapy in the dorsal region and one point anterior to the shoulder. The points posterior to the trunk are, in craniocaudal orientation: cervical facets (midpoint between C1 and C4); scapular enthesis of the levator scapulae; entheses of the lesser and greater rhomboids; supraspinatus point over the suprascapular nerve; and midpoints between the scapular entheses and the thoracic spinous processes, seeking to reach the spinal portion of the accessory nerve and dorsal scapular nerve (three points). The anterior point is located on the coracoid process, seeking to reach the scapular enthesis of the pectoralis minor.

**Figure 7:**
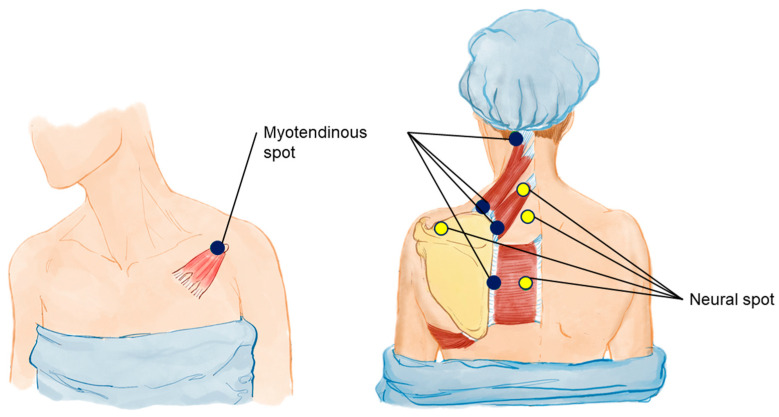
Myotendinous and neural injection spots for UCS. This illustration identifies the specific myotendinous and neural injection spots targeted in the mesotherapy protocol for treating UCS. Yellow dots indicate neural spots, essential for releasing fasciae entrapments, modulating neural inputs and reducing pain. Blue dots highlight additional target areas on the myotendinous junctions of muscles affected by UCS. These targeted injection spots are integral to the 8:1 block treatment approach, aiming to correct muscle imbalances and alleviate CSB pain associated with UCS.

Shortly, we describe a point with a facet focus; four points with a myotendinous focus; and four points with a neural focus. Therefore, this is a mixed neuromyotendinous block (Figure 7).

## 7. Discussion and Description of the Technique

As previously mentioned, the scapula is routinely overlooked in medical evaluation. When it comes to cervicobrachial pathologies, this disparity is even more striking. Since it is clearly neurogenic in origin in only 20% of cases [11], routine evaluation of the scapula is appropriate in this context.

Still little discussed in the literature, levator scapulae and rhomboid tendinopathies can significantly influence neck pain [33,34,35,36]. The biomechanical action of the levator scapulae, with its origin in the transverse processes from C1 to C4, is notable in neck pain. Its non-physiological tension can pull this vertebral block and increase intradiscal pressure throughout the cervical spine, due to hypertonia of varying origins. Similarly, the tension of the rhomboids, with their insertions on the spinous processes, acts as a vertebral pain point of the cervicothoracic transition. Facet degenerations of the cervical spine are confirmed causes of neck pain [37,38,39,40]. Therapeutic intervention in these degenerations has also had very satisfactory results in the literature [39,40,41]; therefore, its inclusion in this protocol is quite sensible.

**Figure 8:**
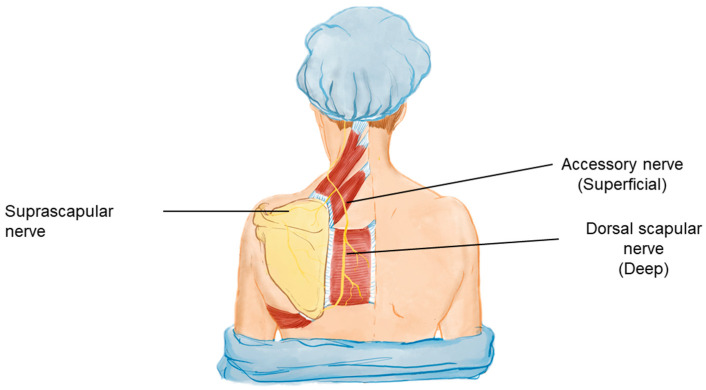
Nerve anatomy relevant to UCS. Schematic representation of the key nerves involved in the pathology of UCS. The labeled nerves include the suprascapular nerve, the accessory nerve (superficial), and the dorsal scapular nerve (deep). These nerves innervate critical muscles that are often affected in UCS, contributing to muscle imbalances, weakness, and pain in the cervicoscapulobrachial region.

Regarding the nerves covered, it is known that these topographies are common points for nerve entrapment. The suprascapular and dorsal scapular nerves with these pathologies are already widely discussed and with interventional effectiveness elucidated in the literature [42,43,44,45]. In order to add to the topography of the blocks and act on a muscle that is very important in the stability of the shoulder girdle, the spinal branch of the accessory nerve can be an excellent consort of the blocks (Figure 8).

Being the only focus of anterior trunk treatment, the pectoralis minor has a very relevant function in this suggested syndrome. Contributing to scapular dyskinesis as a cause or effect, the pectoralis minor causes shortening of its belly and overload of the enthesis in these cases [13,14]. It is worth highlighting the intimate relationship between the tendon of this muscle in the coracoid process and the vascular-nervous bundle that passes through this topography. This complex is composed of the subclavian artery and vein and the brachial plexus. In presentations with myotendinous shortening, there may be a painful syndrome mimicking thoracic outlet syndrome and radiating to the ipsilateral upper limb, such as brachialgia [13,14]. It is, therefore, a good summation within a protocol that aims to encompass the greatest number of diagnostic possibilities of the scapula.

**Figure 9:**
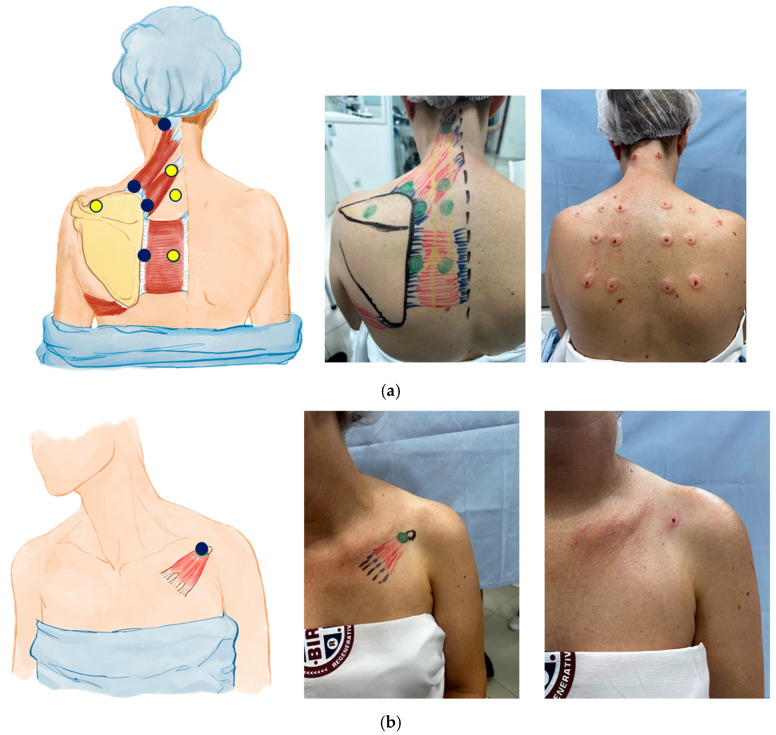
(**a**) Mesotherapy injection protocol for the cervicoscapulobrachial region in UCS, posterior view. (**b**) Anterior view.

Finally, mesotherapy was chosen as a technique due to its practicality and effectiveness. This technique consists of injecting small doses of solutions intradermally. Its action focuses on the activation of stress-induced analgesia during the puncture and the local concentration of the administered medications [46,47,48,49]. The solution standardized by our protocol follows Table 2, with punctures being administered through a 30G needle in a 3 mL syringe at a dose of 0.7 to 1.2 mL of solution, in two or three sets of injections, progressively spreading the target point. Before the final solution, anesthesia is also performed with the same material in the form of mesotherapy with 0.2 mL of 1% lidocaine. The standardized injection points are described in Figure 9a,b.

This set of images shows the conjuncture of schematic figures, in vivo design of anatomy and injection spots and the presentation after treatment with the 8:1 block. Figure 9a represents the posterior spots; Figure 9b represents the anterior spots.

### 7.1. Protocol Description

Each patient received injections using a 30G needle with a 3 mL syringe. The solution comprised dextrose 50% (1.5 mL), procaine 0.7% (4.8 mL), meloxicam 15 mg (1.5 mL), Arnica montana D2 (2.2 mL), N-acetyl-cysteine 100 mg (3 mL), and thiocolchicoside 4 mg (2 mL). The total solution volume was 15 mL, which was divided across nine injection points.

### 7.2. Injection Sites

- **Posterior Points (8):**
  1. Cervical facets (between C1 and C4)
  2. Scapular enthesis of the levator scapulae
  3. Entheses of the lesser and greater rhomboids
  4. Supraspinatus point over the suprascapular nerve
  5. Three additional points between the scapular entheses and thoracic spinous processes targeting the spinal portion of the accessory nerve and dorsal scapular nerve.

- **Anterior Point (1):**

The coracoid process targeting the scapular enthesis of the pectoralis minor.

### 7.3. Dosage and Frequency

Patients received 0.7–1.2 mL of solution per injection point. Treatments were administered once a week for a duration of four to eight sessions.

### 7.4. Anesthesia

A pre-injection anesthetic of 0.2 mL 1% lidocaine was used at each injection site to minimize discomfort.

The proposed intervention frequency is weekly, in cycles of four to eight sessions. As an adjuvant, external shockwave therapy (ESWT) can be used with a myofascial focus on the regional topography addressed in interventional treatment [50].

It is worth highlighting that, with the proposed treatment, there is a tendency for the treated symptoms, both painful and irradiated, to be immediately alleviated. An arthrokinematic realignment of the scapulothoracic joint is also noted in conjunction with symptomatic improvement. Therefore, FHP, previously quite evident, tends to a return of physiological biomechanics, leading the authors to suggest that this is one of the probable causes of UCS as a postural syndrome.

During the application of the 8:1 block protocol, both patients reported immediate pain relief following the treatment sessions, with sustained improvement in scapular mobility and reduction in cervicoscapulobrachial pain. These preliminary observations suggest that the mesotherapy protocol is effective in addressing the underlying muscle imbalances contributing to UCS, although further studies are required to confirm its efficacy. No adverse reactions were reported in the two patients treated with the 8:1 block mesotherapy protocol. However, potential side effects of mesotherapy may include local irritation, allergic reactions, infection, or inflammation at the injection sites. While none of these were observed in our case study, future studies should assess the short-term and long-term safety of this protocol. Proper aseptic techniques and patient monitoring are critical components in reducing the risk of complications.

## 8. Conclusions and Final Considerations

Cervicobrachial pain is a very common complaint in clinical practice. The differential diagnoses for this presentation are varied and do not yet include pathologies that are underdiagnosed, such as scapular tendinopathies and scapular nerve entrapment. Therefore, we propose the term cervicoscapulobrachialgia as a way of bringing attention to the shoulder girdle in the list of pathologies that can lead to these symptoms.

Reaching key points to encompass as many pathologies as possible, the proposed mesotherapy protocol is cheap, feasible and efficient. Respecting the cervicoscapular anatomy and focusing on neutralizing muscular tension points, facet degeneration and nerve entrapment, there is freedom in adding new points or selecting à la carte proposed points.

If the therapeutic target is properly treated, the tendency is for scapular biomechanics to be restored, mischaracterizing FHP as a postural change. Therefore, the SUT of the shoulder elevator muscles is released, allowing, in addition to clinical improvement, quality rehabilitation for the patient. Strength and stretching exercises can then be prescribed with greater efficiency and orthopedic safety, always aiming for the patient’s well-being.

Understanding cervicoscapulobrachial dynamics is extremely important for the treatment of complex upper limb pain syndromes. Since this is an initial proposition, it is still necessary to deepen these propositions and structure new, increasingly complex studies on the theme presented.

As a technical note, this paper aims to introduce the 8:1 block mesotherapy protocol with preliminary clinical observations. However, we recognize the need for future studies to incorporate objective outcome measures, such as the Visual Analog Scale (VAS) for pain assessment, range of motion measurements, and functional mobility tests. These tools will provide quantifiable data to further support the clinical observations presented and validate the protocol’s effectiveness. We acknowledge that the observations in this paper are based on a small sample of patients, and no objective assessment tools, such as pain scales or functional outcome measures, were used. Additionally, there was no long-term follow-up to assess the sustained effectiveness of the treatment.

While the 8:1 block mesotherapy protocol shows promising results in managing cervicoscapulobrachial pain, further research is necessary. Future studies should focus on larger, randomized controlled trials with objective assessment tools, such as pain scales and functional mobility tests. Additionally, long-term safety and comparative studies with existing treatments would help validate and refine the protocol for broader clinical application.

## 9. Author’s Note

As discussed through the topic above, this is a proposal of scapular targeting for treating cervicoscapulobrachial pain. In our clinical practice, these points of injection work as a guide to intervention and are often expanded. For example, the myofascial point in cervical facets may be treated as three different injection points, focusing the area between the C1 and C4 facets. As well, the rhomboid major tendinous point on the scapula border may be treated in two or three points.

Other aspects that must be evaluated are the acromioclavicular joint, and the origin of rhomboids, minor and major, over the spinous processes, mainly the C7 process, which is often painful in this syndromic presentation. In addition, interventions on the occipital nerves over the skull base are a strong addition to this protocol when necessary.

These aspects make the decision of injection points as free as possible for the physician that treats these patients, neglecting or expanding these target points.

## Figures and Tables

**Table 1:** Anatomic Changes and Associated Muscle Imbalances.

Anatomic Change	Tightened Muscles	Weakened Muscles
Shoulder elevation, protraction, and internal rotation	Upper trapezius, levator scapulae, pectoralis minor	Middle trapezius, serratus anterior
Chin anteriorization and neck extension	Levator scapulae, suboccipital muscles, sternocleidomastoid	Scalenes, lower trapezius
Thoracic kyphosis	Pectoralis minor, pectoralis major, sternocleidomastoid	Scalenes, lower trapezius, middle trapezius

**Table 2:** Composition and Volumes of Mesotherapy Components.

Solution Component	Volume	Purpose
Dextrose 50%	1.5 mL	Provides osmotic effect for pain relief
Procaine 0.7%	4.8 mL	Local anesthetic to reduce pain
Meloxicam 15 mg	1.5 mL	Anti-inflammatory agent
Arnica montana D2	2.2 mL	Homeopathic remedy for inflammation
N-acetyl-cysteine 100 mg	3 mL	Antioxidant to reduce oxidative stress
Thiocolchicoside 4 mg	2 mL	Muscle relaxant
**Total Solution**	**15 mL**	**Combined solution for mesotherapy injection**

## Data Availability

No new data generated.
